# “I did it before, so I can do it again(?)”: Recalling success, expectations of future success and the impact of ease-of-retrieval and attributions

**DOI:** 10.1007/s00426-025-02136-x

**Published:** 2025-07-02

**Authors:** Adam Abdulla, Ruth Woods

**Affiliations:** Robert Gordon University, Aberdeen, Scotland

## Abstract

It is widely assumed that recalling past success raises expectations of future success (“expectancy”). However, experimental research investigating that assumption has generated mixed results. The present study examined two (meta)cognitive factors that may play a role during “recall success” interventions: ease-of-retrieval (i.e. the ease/difficulty with which success is recalled) and causal attributions (i.e. the factors to which the success is attributed). Three experiments were conducted with English-speaking adults across the world. After being asked to recall either attraction “success(es)” or attraction “failure(s),” participants reported the extent to which they expected to attract someone in the future (“expectancy”). Results suggest that difficulty in retrieving examples of success and failure to attribute recalled success to stable/general factors have a negative impact on expectancy. Moreover, individuals with low self-perceived mate value are apparently more likely to experience difficulty-in-retrieval and less likely to attribute (attraction) success to stable/general factors. Unless ease-of-retrieval and attributions are addressed, those most in need of an expectancy boost may not benefit from “recall success” interventions.

## Recalling past success and expectations of future success

Alf is unhappy in his love life. He makes little effort to meet potential partners because he does not expect to attract anyone romantically. To what extent would his expectations be raised if he recalled a time when he *did* attract someone romantically? It might be thought that recalling success in a given domain raises expectations of success in that domain. Many authors in the personal (and professional) development literature do assume that recalling success enhances “hope” (Tompkins, 2020), “confidence” (Parsloe & Leedham, 2017), “belief” (Lenson, 2018), perceived “self-efficacy” (Williams, 2024) and “expectations” of future success (Schwindt, 2014). Moreover, the assumption that recalling success enhances expectancy-like variables (e.g. hope, optimism, confidence) lies behind several therapeutic and coaching techniques. For example, solution-focused therapists and coaches encourage individuals who are experiencing a problem to recall “exceptions,” i.e. occasions when they were in fact successful (e.g. Berg & Szabó, 2005). Advocates believe that asking individuals to recall “exceptions” enhances hope, optimism and expectancy (e.g. Parsons, 2009; Reiter, 2010; Winbolt, 2011).

Goal attainment expectancy (i.e. the extent to which people expect to achieve their goals) is crucial for motivation, goal pursuit and mental health. The higher people’s expectancy, the more motivated they are to pursue their goals (e.g. Senko & Hulleman, 2013). In addition, higher expectations of goal attainment are associated with enhanced well-being (e.g. Gamble et al., 2020). Individuals with low expectancy are particularly likely to suffer psychologically if they remain committed to their goals (Brunstein, 1993). If recalling success raises expectancy, both motivation and well-being could then be affected.

Recalling past success may raise expectancy if people rely on an “availability heuristic.” Tversky and Kahneman (1973, p.231) described the “temporary rise in the subjective probability” of an event after one has just witnessed an instance of that event. For example, a woman may momentarily consider a car accident (more) likely if she has just seen a damaged car on the road. A man may have (temporarily) higher expectations of winning the lottery if his neighbour has just shown him a winning ticket. Finally, Alf may (temporarily) have higher expectations of attracting someone romantically if he has just recalled an “attraction success” (i.e. a time when he succeeded in attracting someone). In each case the subjective probability of the event in question is thought to be raised by the example. However, as explained later on, the ease or difficulty with which the example is brought to mind may also be important.

### Asking participants to recall success in experimental research

In experimental research, “recall success” manipulations have apparently had positive effects on expectancy-like variables such as hope (Snyder et al., 1996), perceived self-efficacy (Brown et al., 2016), perceived competence (Austin & Costabile, 2021), perceived social skills and confidence (Wehr, 2010), and expectations of future success (Nelson & Knight, 2010). However, asking participants to recall success does not always have the desired effect. For example, a table provided by Wehr (2010)—Table 2—indicates that students who were asked to recall a single “exception” (i.e. example of success) actually reported *lower* confidence in their ability to manage the problem in future than students asked to recall an example of the problem. Krans et al. (2018) observed increases in mean self-confidence from pre-recall to post-recall in a “recall success” condition. However, an even greater increase was observed in a control condition. Finally, several studies examining the impact of “recall success” interventions on perceived self-efficacy have found little evidence of positive effects (e.g. Abdulla, 2021; Solms et al., 2022; Vanlede et al., 2009). Many explanations may be offered for the negative results. The present investigation focuses on two (meta)cognitive factors that may be relevant: ease-of-retrieval (i.e. the ease/difficulty with which success is recalled) and causal attributions (i.e. the factors to which success is attributed).

### Ease-of-retrieval when attempting to recall success and failure

#### Ease-of-retrieval, self-esteem and self-perceived mate value

**Fig. 1:**
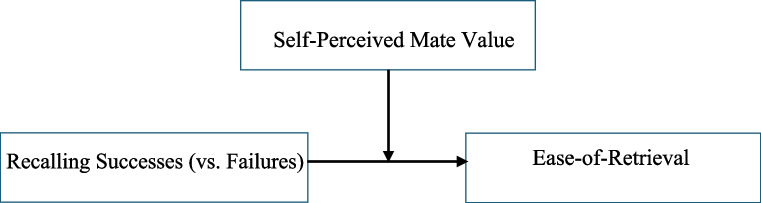
The hypothesised impact of self-perceived mate value on the relative ease of recalling attraction successes (vs. Failures)

Dysphoric individuals and individuals with low self-esteem often find it more difficult to recall success and more difficult to recall positive (than negative) autobiographical episodes (Christensen et al., 2003; Köszegi et al., 2022; Matsumoto & Mochizuki, 2017; Smith & Petty, 1995). Moreover, Demiray and Janssen (2015) found that participants with lower levels of self-esteem felt psychologically more distant from positive memories and rehearsed these memories less frequently than participants with higher self-esteem. In the context of attraction, a relevant facet of self-esteem is *self-perceived mate value.* Individuals with low self-perceived mate value consider themselves to have low value as potential “mates” (Fisher et al., 2008). General self-esteem and self-perceived mate value are positively correlated (Goodwin et al., 2012). Individuals with low self-perceived mate value may find it easier to recall (attraction) *failures* than successes because failures are more consistent with their self-schema. On the other hand, high self-perceived mate value may facilitate retrieval of attraction “successes”. In other words, whether it is easier to recall attraction successes or failures depends on an individual’s self-perceived value, as illustrated in Fig. 1.

The following hypotheses were formulated on the basis of the foregoing considerations:

- **H1:** When self-perceived mate value (SMV) is low, individuals find it more difficult to recall examples of attraction success than examples of attraction failure
- **H2**: When individuals are attempting to recall (attraction) success, the higher their self-perceived mate value, the easier the retrieval
- **H3**: When participants are attempting to recall (attraction) failure, the lower their self-perceived mate value, the easier the retrieval.

#### The effect of ease-of-retrieval on expectancy

Suppose that Alf finds it difficult to recall a time when he attracted someone romantically. On the other hand, suppose that Bob finds it easy to recall an “attraction success”. All else being equal, Alf may then have lower expectations of attracting someone romantically (i.e. lower attraction expectancy) than Bob. Support for this hypothesis derives from studies on ease-of-retrieval, which is often associated with the “Availability Heuristic” (e.g. Schwarz et al., 1991). It is useful, however, to distinguish between “availability” on the one hand and “ease-of-retrieval” on the other (MacLeod & Campbell, 1992; Tulving & Pearlstone, 1966). Alf may (eventually) recall an example of success, which means that such an example is “available.” However, if he struggles to bring the example to mind, then he experienced some difficulty-in-retrieval.

Many studies suggest that “when forming a judgment people tend to rely less on retrieved information when the retrieval [feels] difficult as compared to easy.” (Wänke, 2013, p.151). MacLeod and Campbell (1992) asked participants to recall a time when they had experienced a particular event, e.g. a mutual attraction. The researchers measured the time taken by each participant to recall a relevant example. The researchers also measured expectancy by asking participants to judge how likely it was that they would experience such an event in the next six months. The longer participants took to recall an example of an event the less likely they considered its recurrence. Together with other findings, this was taken as evidence that ease-of-retrieval affects expectancy when individuals are attempting to recall examples of events.

#### Ease-of-retrieval and confounding variables

Associations between ease-of-retrieval and expectancy-like variables need to be treated with caution. Researchers must consider the possibility of confounding (Abdulla & Woods, 2022). Suppose, for example, that difficulty in recalling success is negatively associated with expectancy. It may be tempting to conclude that difficulty-in-retrieval is lowering expectancy. However a third variable—age—may be responsible for the association. Several studies have found a negative association between age and expectancy-like variables (Abdulla, 2023; Bühler et al., 2019; Durbin et al., 2019; Giltay et al., 2006). Some aspects of autobiographical recall (e.g. the retrieval of episodic details) also decline with age (Hernandez et al., 2024). Suppose that individuals experiencing difficulty-in-retrieval (when attempting to recall success) subsequently report lower expectancy. This *may* indicate a negative effect of retrieval difficulty on expectancy. On the other hand, it may simply reflect old age. Researchers should therefore investigate whether ease-of-retrieval is associated with expectancy even when age is held constant. 

Another variable that should be held constant is task- or domain-specific self-esteem. As already noted, individuals with low self-esteem often find it more difficult to recall success (e.g. Christensen et al., 2003; Köszegi et al., 2022). Self-esteem and expectancy are often positively associated (e.g. Abel, 1996). Kavanagh et al. (2010) found that higher self-esteem was associated with higher “mating aspirations”—a variable close to “attraction expectancy”. Moreover, task- or domain-specific self-esteem is even more closely associated with expectancy than generalised self-esteem (Hollenbeck & Brief, 1987). In the present context, domain specific self-esteem is self-perceived mate value and one hypothesis is as follows:

- **H4**: Self-perceived mate value has a positive effect on (attraction) expectancy.

According to H2, low self-perceived mate value is associated with difficulty-in-retrieval when individuals attempt to recall success. H4 implies that low self-perceived mate value is also associated with lower (attraction) expectancy. If these hypotheses are correct, then any association between ease-of-retrieval and expectancy may be due to another variable: self-perceived mate value.

**Fig. 2:**
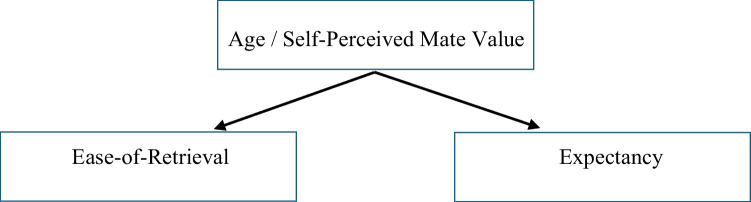
No relationship between ease-of-retrieval and expectancy with age/self-perceived mate value as a confounder

In short, if age and self-perceived mate value are genuine confounders there may be no effect of ease-of-retrieval on expectancy. Figure 2 illustrates this possibility.

In order to discover whether ease-of-retrieval affects expectancy, both age and self-perceived mate value should be controlled. The following hypotheses were tested in the present study:

- **H5**: When individuals of the same age and self-perceived mate value are recalling (attraction) success, the greater the difficulty-in-retrieval, the lower their expectancy
- **H6:**When individuals of the same age and self-perceived mate value are recalling (attraction) failure, the greater the difficulty-in-retrieval, the higher their expectancy.

**Fig. 3:**
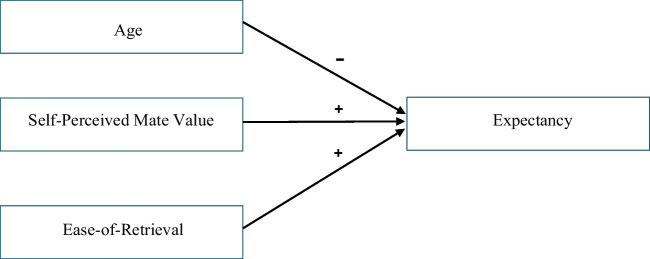
Independent (Hypothesised) Effects on expectancy of age, self-perceived mate value and ease-of-retrieval when individuals attempt to recall success

Support for H5 in turn supports the hypothesis that ease-of-retrieval *affects* expectancy when individuals attempt to recall success. A direct effect of ease-of-retrieval on expectancy is illustrated in Fig. 3, which also illustrates negative and positive effects (on expectancy) of age and self-perceived mate value, respectively. Figure 3 contrasts with Fig. 2, in which there is no arrow from ease-of-retrieval to expectancy.

### Past Success and the stability and generality of causal factors

Whether recalling success enhances expectancy may also depend on attributions. According to Weiner’s attribution theory, the causes of success have three key properties: (i) perceived stability, (ii) perceived controllability, and (iii) perceived locus (e.g. Weiner, 1985). Weiner argues that it is perceived *stability* that determines expectancy (e.g. Weiner, 1985, 2012, 2018). Stable causes of success include characteristics that are consistent over time, e.g. personality. Unstable causes are temporary or changeable, e.g. mood or luck. Suppose that Alf and Bob both recall times when they attracted someone. Alf attributes his success to an unstable factor (“It was pure chance”), whereas Bob attributes his to a stable factor (“I have an attractive personality”). All else being equal, Bob should have greater attraction expectancy than Alf. Several studies have indeed found that the perceived stability of the cause of a success is positively associated with expectancy (e.g. Meyer, 1980; Nickel & Spink, 2010; Van Overwalle et al., 1995; Weiner, 1986).

Expectancy should however also be influenced by the perceived *generality* of the cause of success. Perceived stability is thought to influence temporal aspects of expectancy whereas generality (or “globality”) influences cross-situational expectancies (e.g. Weiner, 2012). Imagine two women—Claire and Dawn—who each attract a man at a party. For Dawn, the cause of success is specific to the man at the party: “He shared my passion for candle-making.” Claire, on the other hand, attributes her success to a general “fact”: “Men like my sense of humour.” All else being equal, Claire should have higher (attraction) expectancy than Dawn.

It may therefore be hypothesised that individuals who attribute successes to factors that are both stable and general (or “global”) have higher expectancy than those who fail to attribute successes to such factors. Some research supports that hypothesis (e.g. Coffee & Rees, 2008; Cropley & Macleod, 2003). Once again, however, attention to confounding variables is important. Cohen et al. (1989) found that low self-esteem was associated with attributions of success to external, unstable and specific factors whereas high self-esteem was associated with attributions to internal, stable and global factors. Other research similarly suggests that those with low self-esteem are more likely to attribute success to unstable factors (e.g. Fielstein et al., 1985; Hesketh, 1984; Stake, 1990; Weiss et al., 1990) and less likely to attribute success to general factors than those with high self-esteem (Zunick et al., 2015). As noted, the domain-specific form of self-esteem in the present study is self-perceived mate value and one hypothesis is therefore as follows:

- **H7**: Individuals with higher self-perceived mate value are more likely to attribute attraction success to factors with (at least limited) generality and stability than individuals with lower self-perceived mate value.

If H7 is correct, then individuals who attribute recalled success to specific and unstable factors may also be low in self-perceived mate value. As explained, low self-perceived mate value is likely to be associated with lower attraction expectancy (H4). An association between attributions and expectancy may therefore be due to a third variable: self-perceived mate value. In order to disentangle the effect of attributions from the effect of self-perceived mate value, the latter must somehow be controlled.

#### “Stability” and “Generality” on a continuum

H7 uses the expression “(at least limited).” It is important to realise that “stability” and “generality” exist on a continuum, as illustrated in Table 1 (Weiner, personal communication). The columns capture three levels of stability—1) Unstable, 2) Limited Stability, and 3) Stable—and the rows capture three levels of generality—1) Highly specific, 2) Limited Generality, and 3) Global. “Limited Stability” and “Limited Generality” are intermediate between the two poles of each dimension. The responses in each cell in Table 1 are hypothetical explanations for success in attracting a man. The shaded cells capture attributions to factors of at least limited stability *and* at least limited generality. The unshaded cells capture attributions to factors that are either highly specific or unstable. People may also fail to attribute successes to any factors at all (e.g. “I don’t know how I did it.”).

**Table 1:**
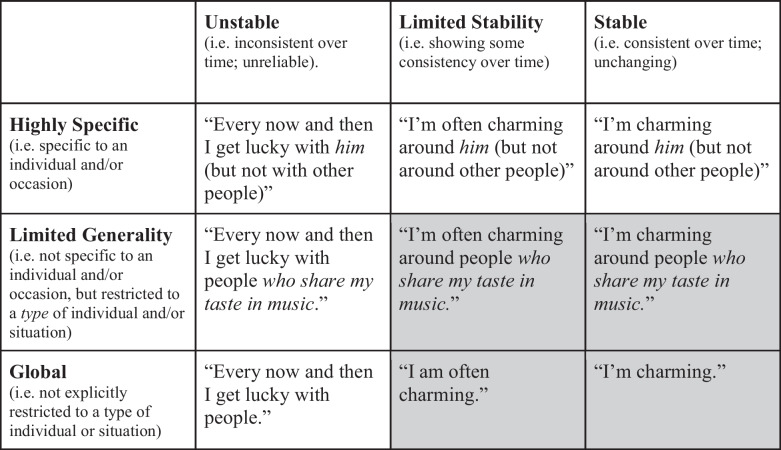
Hypothetical attributions of success in attracting a man

If attribution theorists are correct, then, ceteris paribus*,* individuals whose attributions of success fall into the shaded cells in Table 1 (i.e. individuals attributing success to factors of at least limited stability and generality) should have higher expectancy than individuals whose attributions of success fall into the unshaded cells (i.e. individuals attributing success to unstable or highly specific factors). With potential confounders taken into account, the hypothesis is as follows:

- **H8**: When individuals of the same age and self-perceived mate value experience the same ease/difficulty in recalling (attraction) success, those who attribute successes solely to factors of at least limited stability and generality have higher expectancy of future success than individuals who fail to attribute successes (solely) to such factors.

### Asking individuals who are struggling in a domain to recall success in that domain

In many forms of therapy, coaching and self-help, individuals struggling in a particular domain are asked to recall success in that domain. For example, Sharma (2009, p.213) offers the advice: “Recall a time when you succeeded in your struggle.” However, there are reasons for thinking that individuals struggling in a particular domain may not benefit from a “recall success” intervention. First, if such individuals have frequently experienced failure in the domain, then they may find it difficult to recall success. Second, lack of success in a domain lowers self-esteem in that domain. For example, lack of success in attracting others lowers self-perceived mate value (e.g. Kavanagh et al., 2010; Zhang et al., 2015). According to H2, low self-perceived mate value leads to difficulty-in-retrieval when individuals attempt to recall attraction success. If difficulty-in-retrieval lowers expectancy (see H5), then attempting (and struggling) to recall success may do more harm than good.

In addition, if lack of success in a domain has lowered self-esteem in that domain, then attributions may also be maladaptive. As noted, low self-esteem is often associated with a tendency to attribute success to unstable and specific factors (e.g. Cohen et al., 1989). Individuals with low self-esteem or low self-perceived mate value may therefore fail to make the sorts of attributions required by “recall success” interventions (see H7 and H8).

In short, asking individuals who are struggling in a domain to recall success in that domain may fail to raise expectancy. In addition, those with *high* self-esteem or high self-perceived mate value should already be high in expectancy (Hollenbeck & Brief, 1987; Kavanagh et al., 2010). There may then be little room for expectancy improvement—i.e. a ceiling effect (Gist & Mitchell, 1992; Karl et al., 1993). If so, then a “recall success” intervention may be ineffective with individuals both high and low in self-perceived mate value.

## The present study

The present study was advertised to individuals who were “struggling to attract the right person.” It was approved by the Ethics Committee at Robert Gordon University. Participants were asked to recall attraction successes or failures. The key dependent variable was attraction expectancy, i.e. the extent to which participants expected to attract someone in the future. Two (meta)cognitive factors were closely examined: ease-of-retrieval and causal attributions. The present study is, to the authors’ knowledge, the first to investigate simultaneously the effects of these factors on posttest expectancy while controlling for key confounders, e.g. age and self-perceived mate value. Moreover, unlike almost all other studies of “recall success” manipulations, the present investigation was not limited to students in a particular location. Rather, participants were adults of all ages around the English-speaking world. They were recruited through Prolific (a large, diverse online pool of participants). Douglas et al. (2023) found that Prolific generated data of a higher quality than MTurk, Qualtrics and an undergraduate student sample. Demographic prescreeners were applied to ensure that participants met the following criteria: 1) They were single; 2) They spoke English as their first language; 3) They had a 100% approval rating on Prolific. Prospective participants read a short description of the study and those who wished to participate provided informed consent. For the sake of confidentiality participants on Prolific are assigned a unique Participant ID (a string of numbers and letters) and do not share their identity (i.e. their names) with researchers.

### Sample size planning

A priori power analysis and sample size planning were challenging for the present study. First, previous studies of “recall success” interventions have yielded very different effect size estimates, Cohen’s *d* ranging from 0.08 (Abdulla, 2021) to 1.16 (Nelson & Knight, 2010). Second, many of the hypothesised effects are moderation or conditional effects. A priori power analysis in the context of such effects is unlikely to be a fruitful endeavour (Hayes, 2022). Nevertheless, if a between-condition difference is moderately large in the population (i.e. *d* = 0.5), then 64 participants per condition are estimated to be sufficient to achieve 0.80 power (*GPower*). Approximately twice as many participants were recruited for each of the experiments. Power should therefore have been adequate to detect a moderately large effect of recalling success if any such effect existed. However, more attention was paid to the consistency of results and estimated sizes of effects than to statistical significance.

### Analytical strategy

#### Data inclusion and exclusion

Including data from participants who do not recall the specified number of examples makes it difficult to interpret results (Wänke, 2013). Specifically, a low expectancy score might stem not from experiencing difficulty-in-retrieval but from recalling fewer examples. Data from participants who did not retrieve the specified number of examples were therefore excluded. 

#### Estimating the effect of ease-of-retrieval on expectancy

In the present study, the effect of ease-of-retrieval on expectancy was estimated in two regressions. In the first regression, expectancy of participants in both conditions (“recall success” and “recall failure”) was the dependent variable. In the second, expectancy in the “recall success” condition was regressed on both ease-of-retrieval and attributions (as well as the covariates—age and self-perceived mate value).

#### Analysis of qualitative data: perceived stability and generality

After recalling attraction success, participants in the “recall success(es)” condition were asked the following question: “What does it say about you that you were able to attract that person[those people]?” Deductive content analysis was used to determine the stability and generality of the causal factors mentioned in responses. The authors followed the process outlined by Krippendorff (2018) and summarised by McKibben et al (2022).

##### The Stability Dimension

*Stable* factors were defined as factors typically considered to be consistent over time, e.g. being “funny.” A factor was deemed to be of *limited stability* if the participant mentioned a stable factor but included a temporal qualifier, e.g. “I’m *normally* funny”. Finally, *unstable* factors were defined as factors typically considered to fluctuate over time such as luck and mood (e.g. Weiner et al., 1978) and “unusual exertion” (Weiner, 2018, p.5). The stability dimension therefore had three levels: 1) Stable, 2) Limited Stability, and 3) Unstable. A factor was said to be of “at least limited stability” if either of the first two levels applied.

##### The Generality Dimension

*Global* factors were defined as factors described without any explicit restriction on the types of individuals with whom or situations in which they applied (“I’m charming”). A factor was deemed to be of *limited generality* if the description implied that the factor applied only with certain types of individuals or in certain types of situations (“I’m charming *around people who like animals”*). Finally, *highly specific* causal factors were defined as factors (i) specific to the individual(s) in the recalled example(s) (“*She* finds me charming”), or (ii) specific to the occasion, interaction or relationship between the participant and the individual(s) (“We happened to share an interest in Cuban music”). The generality dimension therefore also had three levels: 1) Global, 2) Limited Generality, and 3) Highly Specific. A factor was said to be of “at least limited generality” if either of the first two levels applied.

Each participant’s response was assigned to one of the following categories:

1. All causal factors mentioned in the response have at least limited stability and generality
2. The response mentions an unstable or highly specific causal factor
3. The response does not mention any causal factor
4. The stability and generality of factors mentioned in the response are unclear and the response does not fall readily into any of the other categories.

The four categories were established on an a priori basis and definitions of the key terms (i.e. “stable,” “unstable,” etc.) were based on the work of Weiner (e.g. Weiner, 1985, 2021). A codebook was created to provide guidelines for determining the stability and generality of causal factors mentioned by participants. The guidelines were based on the work of Weiner and others who have studied attribution (e.g. Zullow et al., 1988). The coding scheme was piloted as per the recommendations of McKibben et al. (2022).

## Experiment 1

### Methods

#### Participants

Individuals were invited to sign up for the study via Prolific if they were “struggling to attract the right person.” Initially, 250 individuals were recruited and randomly assigned to a “recall success” *(n* = 125) or “recall failure” condition *(n* = 125). In the “recall success” condition 97.6% (122 individuals) and in the “recall failure” condition 96.8% (121 individuals) completed the study. Those who did not complete the study did not submit any data and were therefore excluded from analyses. Ages ranged from 18 to 75 (M = 34.5, SD = 12.3). Reported nationalities included British (71%), North American, i.e. a citizen of the US or Canada (9%), South African (6%), Australian or New Zealander (6%) and various other nationalities, e.g. Zimbabwean, Nigerian and Indian (8%). Of the 242 participants (99.6%) who reported their gender, 56% selected “male” (136 participants), 43% selected “female” (103 participants) and 1% (3 participants) selected “other.”

#### Procedure

Participants were initially asked for demographic information and presented with self-perceived mate value questions. Participants in the “recall success” condition were then asked to recall and describe a time when they had attracted somebody whom they wanted to attract. They were then asked: “What does it say about you that you were able to attract that person?” Questions of this sort are asked in many forms of coaching, counselling and therapy (e.g. Beck, 2021; Brown & Augusta-Scott, 2006; O'Connell et al., 2013). Participants in the “recall failure” condition were asked to recall and briefly describe a time when they had *not* attracted somebody whom they wanted to attract. They were then asked: “What do you think prevented you from attracting that person?” Questions of this sort are often asked in problem-focused coaching/counselling interventions (e.g. Dryden, 2019). Participants in both conditions were then asked to respond to questions assessing attraction expectancy and ease-of-retrieval.

### Measures

#### Self-perceived mate value

The “Mate Value Scale” (Edlund & Sagarin, 2014) was used to measure self-perceived mate value (SMV). Edlund and Sagarin (2014) report evidence of both convergent and discriminant validity for this measure and high reliability of the data generated. The first question is: “Overall, how would you rate your level of desirability as a partner on the following scale?” The scale for each question ranged from 1 to 7, higher scores indicating higher SMV. One of the questions uses the phrase “the opposite sex.” In order to accommodate all sexual orientations, that phrase was replaced by “the sex that you’re trying to attract*”*. Estimated reliability was extremely high (*α* = 0.91).

#### Expectancy

Expectancy was measured by means of the instrument developed by Abdulla (e.g. Abdulla, 2023; Abdulla & Woods, 2022). The instrument has been used in numerous contexts including interpersonal relationships (Abdulla, 2023). Obtained scores have been found to correlate with goal commitment (e.g. Abdulla, 2024), providing evidence of construct validity. The first question was: “On a scale from 0 to 10, how likely is it that you will (at some point) attract the sort of person you want to attract?” The scale for each question ranged from 0 to 10, higher scores indicating higher expectancy. Estimated reliability was extremely high (*α* = 0.93).

#### Ease-of-retrieval

Ease-of-retrieval was measured by means of the instrument used by Abdulla and Woods (2022), who report evidence of factorial and construct validity. The first question was: “On a scale from 1 to 7, how hard was it to think of a time when you attracted[did not attract] somebody you wanted to attract?” The scale for each question ranged from 1 to 7, *lower* scores indicating greater ease-of-retrieval. As in the study reported by Abdulla and Woods (2022), lower scores on this measure were obtained when participants were asked to think of fewer examples, further supporting construct validity. Estimated reliability was very high (*α* = 0.87).

### Results

Means and standard deviations of measured variables in each condition are presented in Table 2.

**Table 2:** Means and standard deviations of measured variables in experiment 1

	“Recall Success”		“Recall Failure”	
	M	SD	M	SD
Self-Perceived Mate Value	3.84	1.15	3.89	1.08
(Attraction) Expectancy	4.27	1.73	4.29	1.78
Ease-of-retrieval	3.79	1.35	3.26	1.32

In the “recall success” condition 111 participants (91%) and in the “recall failure” condition 111 participants (92%) were able to recall an example.

#### Self-perceived mate value and ease-of-retrieval

According to H1, when self-perceived mate value (SMV) is low, individuals find it more difficult to recall success than failure. In order to test H1, ease-of-retrieval was regressed on condition, SMV, the product of condition and SMV (the interaction term) and age. The coefficient for the interaction term was statistically significant: *b* = −0.34 [−0.65, −0.03],* t* = 2.14, *p* = 0.033. The Johnson-Neyman technique was used to probe the interaction. When SMV was lower than 4.09, participants experienced (statistically) significantly greater difficulty-of-retrieval in the “recall success” than in the “recall failure” condition. Over half of the participants (57%) had SMV scores lower than 4.09. At one standard deviation below the mean of the SMV distribution (SMV = 2.74), ease-of-retrieval was estimated to be 0.81[0.32,1.30] of a point lower in the “recall success” than in the “recall failure” condition (*t* = 3.26, *p* = 0.001)—a moderately large effect. H1 was therefore supported.

According to H2, self-perceived mate value should be negatively associated with difficulty-of-retrieval (i.e. positively associated with *ease*-of-retrieval) in the “recall success” condition. The observed association was consistent with H2 and very close to statistical significance: *b* = −0.19 [−0.40,0.02],* t* = 1.78, *p* = 0.077. According to H3, self-perceived mate value should be positively associated with difficulty-of-retrieval in the “recall failure” condition. The observed association was consistent with H3, albeit not statistically significant: *b* = 0.15 [−0.08, 0.37],* t* = 1.28, *p* = 0.203.

#### The overall effect of condition on expectancy

Expectancy was regressed on a dummy variable coding condition (“Recall success” = 1; “Recall failure” = 0) and the two covariates (age and self-perceived mate value). The effect of recalling success was estimated to be positive but was very small—approximately one-tenth of a point on a 0 to 10 scale—and not statistically significant: *b* = 0.13 [−0.19, 0.46],* t* = 0.80, *p* = 0.425.

#### Self-perceived mate value, age, ease-of-retrieval and expectancy

In order to test H4-H6, expectancy was regressed on ease-of-retrieval, condition, the product of ease-of-retrieval and condition (the interaction term), self-perceived mate value and age. According to H4, self-perceived mate value has a positive effect on attraction expectancy. Supporting H4, the coefficient for self-perceived mate value was positive and large: *b* = 1.03 [0.88, 1.17],* t* = 13.92, *p* > 0.0001. Age was negatively associated with expectancy: *b* = −0.04 [−0.05, −0.02],* t* = 5.46, *p* < 0.0001.

The interaction term was statistically significant: *b* =—0.34 [−0.59,−0.09],* t* = 2.71, *p* = 0.007. According to H5, when individuals of the same age and self-perceived mate value are attempting to recall success, the greater the difficulty-in-retrieval the lower the expectancy. In the “recall success” condition, greater difficulty-of-retrieval was indeed associated with lower expectancy: *b* =—0.13 [−0.31, 0.05],* t* = 1.42, *p* = 0.156. The estimated effect was however small (just over one-tenth of a point on a 0–10 scale) and not quite statistically significant. According to H6, when individuals of the same age and self-perceived mate value are attempting to recall failure, the greater the difficulty-in-retrieval the *higher* the expectancy. In the “recall failure” condition, greater difficulty-of-retrieval was indeed associated with higher expectancy: *b* = 0.21 [0.04, 0.38],* t* = 2.43, *p* = 0.016. The estimated effect was again small (approximately one-fifth of a point on the 0–10 scale).

#### Qualitative data and causal attributions

Qualitative responses in the “recall success” condition were assigned to one of the four attribution categories on the basis of the stability and generality of the factors mentioned. The authors assigned 95.5% of the responses (106 out of 111) to the same category (Cohen’s κ = 0.93 [0.86,0.99]). Table 3 records the number of responses assigned to each category after the five discrepancies were resolved.

**Table 3:** The number of responses assigned to each attribution category in experiment 1

Category	Number of Responses
1. All causal factors mentioned in the response have at least limited stability and generality	52
2. The response mentions an unstable or highly specific causal factor;	42
3. The response does not mention any causal factor	16
4. The stability and generality of factors mentioned in the response are unclear and the response does not fall readily into any of the other categories	1

In order to test H7 and H8, two “attribution groups” were formed. Attribution group 1 included responses that fell into category 1 (“All causal factors mentioned in the response have at least limited stability and generality”). Attribution group 2 included responses that fell into category 2 (“The response mentions an unstable or highly specific causal factor”) or category 3 (“The response does not mention any causal factor”). As will be observed from Table 3, 52 responses fell into attribution group 1 and 58 responses fell into attribution group 2. Table 4 displays examples of responses and illustrates the process of categorisation and grouping.

**Table 4:** Examples of participant responses in experiment 1 with categorisation and attribution groups

Participant Response	Category	Attribution Group
I think I am very good at flirting and have an above average appearance	1—All causal factors mentioned in the response have at least limited stability and generality	1
I think it was just purely luck. I do not think I am good at reading people so it was kind of hard to tell if she liked me or not. So I think I was lucky to be able to get into a relationship with her	2—The response mentions an unstable or highly specific causal factor;	2
I’m unsure	3—The response does not mention any causal factor	2

#### Self-perceived mate value and causal attributions

According to H7, individuals with higher self-perceived mate value are more likely to attribute attraction success to factors of at least limited generality and stability than individuals with lower self-perceived mate value. In order to test H7, “attribution group” was regressed on self-perceived mate value (SMV) in a binary logistic regression. The coefficient for SMV was positive and extremely close to statistical significance: *b* = 0.32[−0.01,0.65],* p* = 0.069. Thus, an increase in self-perceived mate value was associated with an increase in the probability of attributing success solely to factors of at least limited stability and generality, which supports H7. More specifically, the odds of making such an attribution were estimated to increase by a factor of 1.37 as self-perceived mate value increases by one point.

#### The effect of attributions on expectancy

According to H8, when individuals of the same age and self-perceived mate value experience the same ease/difficulty in recalling (attraction) success, those who attribute successes solely to factors of at least limited stability and generality have higher expectancy of future success than individuals who fail to attribute successes (solely) to such factors. In order to test H8, expectancy in the “recall success” condition was regressed on age, self-reported mate value (SMV), ease-of-retrieval and attribution group. Participants who attributed success solely to factors of at least limited stability and generality (group 1) were estimated to be over three-quarters of a point higher on the 0–10 expectancy scale than participants who failed to attribute success (solely) to such factors (group 2): *b* = 0.78[0.33, 1.23],* t* = 3.46, *p* < 0.001—a moderately large effect. H8 was therefore supported. In this regression, difficulty-in-retrieval was estimated to have a small, negative effect on expectancy: *b* = −0.17[−0.34, 0.01],* t* = 1.91, *p* = 0.059. This lends further support to H5.

### Brief discussion

In Experiment 1, there was little to suggest that asking participants to recall success (rather than failure) had a meaningful overall effect on expectancy. On the other hand, the hypotheses were generally supported. For participants with low self-perceived mate value, it appeared to be more difficult to recall successes than failures. This finding is especially important given that almost 60% of participants had self-perceived mate value scores lower than the midpoint on the scale. Difficulty-in-retrieval was associated with lower expectancy in the “recall success” condition (although the association was not quite statistically significant) and higher expectancy in the “recall failure” condition. Attributions of success to factors of at least limited stability and generality appeared to have a positive effect on expectancy.

Participants in Experiment 1 were asked to recall only a single example of success or failure. The number of examples may be important. In the second experiment reported by Wehr (2010), participants who recalled just one “exception” (i.e. example of success) actually reported *lower* confidence in dealing with the problem in future than participants who recalled one example of the problem. However, participants who recalled *five* “exceptions” reported greater confidence than participants who recalled five examples of the problem. Unsurprisingly, though, participants found it more difficult to recall five examples.

On the one hand, therefore, asking individuals to recall success may have a more positive effect on expectancy if they are asked to recall multiple examples. On the other hand, the more examples that participants are asked to recall, the more difficulty they will experience in retrieval. The effects of ease-of-retrieval are thought to be greatest when the ease/difficulty is particularly salient (Hansen & Unkelbach & Greifeneder, 2013; Wänke, 2013; Wänke, 2013). Recalling *multiple* successes should be much more difficult than recalling a single success, especially if one is struggling in the domain. Difficulty of retrieval should then be more salient and expectancy may then be more negatively affected. Participants in Experiment 2 were therefore asked to recall multiple examples of success (or failure).

## Experiment 2

### Methods

#### Participants

The recruitment process and inclusion criteria were the same as in Experiment 1. Initially, 270 individuals were recruited. Individuals were randomly assigned to a “recall success” *(n* = 135) or a “recall failure” *(n* = 135) condition. In each condition, 121 individuals (90%) completed the intervention. Those who did not complete the study did not submit any data and were therefore excluded from analyses. Ages ranged from 19 to 67 (M = 34.0; SD = 11.7). Reported nationalities included British (77%), North American (9%), Australian or New Zealander (5%), South African (4%) and various other nationalities, e.g. Zimbabwean, French and Chinese (5%). Of the 239 participants (99%) who stated their gender, 122 51% (122 participants) selected “female”, 47% (113 participants) selected “male” and 2% (4 participants) selected “other”.

#### Procedure

The procedure in Experiment 2 was identical to the procedure in Experiment 1 except in the following respects. Participants were asked to recall *three* successes or failures and the expression “those people” replaced “that person.” In previous research on ‘recall success’ interventions, the number of examples has varied. For example, Wehr (2010) asked participants to recall *five* examples of success whereas Fuller et al. (2013) asked participants to recall *three* or *nine* examples (depending on the experimental condition). The lower number (*three*) was selected for the present study given that participants were struggling in the relevant domain and could not be expected to recall five or nine successes.

### Measures

Estimated reliability was once again very high for self-perceived mate value (*α* = 0.87); expectancy (*α* = 0.94) and ease-of-retrieval (*α* = 0.91), all of which were measured by means of the instruments used in Experiment 1.

### Results

Means and standard deviations of measured variables in each condition are presented in Table 5.

**Table 5:** Means and standard deviations of measured variables in Experiment 2

	“Recall Success”		“Recall Failure”	
	M	SD	M	SD
Self-Perceived Mate Value	3.99	1.07	3.73	1.02
(Attraction) Expectancy	4.21	1.84	4.23	1.71
Ease-of-retrieval	4.38	1.38	4.06	1.33

In the “successes” condition 96 participants (79%) and in the “failures” condition 85 participants (70%) were able to recall three examples.

#### Self-perceived mate value and ease-of-retrieval

When ease-of-retrieval was regressed on condition, self-perceived mate value, the product of condition and self-perceived mate value, and age, the interaction was extremely close to statistical significance: *b* = −0.33 [−0.69, 0.03],* t* = 1.81, *p* = 0.072. When self-perceived mate value (SMV) was lower than 3.59, retrieval was estimated to be (statistically) significantly more difficult in the “recall success” than in the “recall failure” condition. Approximately 40% of participants had SMV scores lower than 3.59. At one standard deviation below the mean of the SMV distribution (SMV = 2.86), ease-of-retrieval was estimated to be 0.64 [0.10,1.17] of a point lower in the “recall success” than in the “recall failure” condition (*t* = 2.36, *p* = 0.019)—a moderately large effect. There was therefore further support for H1 (individuals find it more difficult to recall success than failure when self-perceived mate value is low).

Self-perceived mate value was again negatively associated with difficulty-of-retrieval in the “recall success” condition: *b* = −0.25 [−0.49, −0.01],* t* = 2.04, *p* = 0.042. There was therefore further support for H2. Self-perceived mate value was again positively associated with difficulty-of-retrieval in the “recall failure” condition, although the estimated effect was small and not statistically significant: *b* = 0.08 [−0.19, 0.35],* t* = 0.61, *p* = 0.546. The sign of the estimated effect was however consistent with H3.

#### The overall effect of condition on expectancy

The overall effect on expectancy of recalling success was estimated to be all but zero: *b* = −0.001 [−0.36, 0.36],* t* = 0.01, *p* = 0.994.

#### Self-perceived mate value, age, ease-of-retrieval and expectancy

Expectancy was regressed on ease-of-retrieval, condition, the product of ease-of-retrieval and condition (the interaction term), self-perceived mate value and age. The estimated effect of self-perceived mate value on attraction expectancy was again positive and large: *b* = 0.97 [0.80, 1.14],* t* = 11.15, *p* > 0.0001. There was therefore further support for H4. Age was again negatively associated with expectancy: *b* = −0.04 [−0.05, −0.02],* t* = 5.06, *p* > 0.0001.

The sign of the coefficient for the interaction term was the same as in Experiment 1 although not quite statistically significant: *b* = −0.22 [−0.50, 0.06],* t* = 1.57, *p* = 0.117. In the “recall success” condition, greater difficulty-of-retrieval was associated with (statistically) significantly lower expectancy: *b* =—0.23 [−0.42, −0.03],* t* = 2.33, *p* = 0.021. There was therefore further support for H5. In the “recall failure” condition, the association between ease-of-retrieval and expectancy was extremely small and far from statistical significance: *b* = −0.003 [−0.21, 0.20],* t* = 0.02, *p* = 0.984. There was therefore little support for H6.

#### Qualitative data and causal attributions

The authors assigned 93.8% of participants’ responses (90 out of 96) to the same attribution category (Cohen’s κ = 0.90 [0.82,0.99]). Table 6 records the number of responses assigned to each category after the discrepancies were resolved.

**Table 6:** The Number of Responses Assigned to Each (Attribution) Category in Experiment 2

Category	Number of Responses
1. All causal factors mentioned in the response have at least limited stability and generality	57
2. The response mentions an unstable or highly specific causal factor;	29
3. The response does not mention any causal factor	10
4. The stability and generality of factors mentioned in the response are unclear and the response does not fall readily into any of the other categories	0

As may be observed from Table 5, 57 responses fell into attribution group 1 (“All causal factors mentioned in the response have at least limited stability and generality”) and 39 responses fell into attribution group 2 (“The response mentions an unstable or highly specific causal factor;” or “The response does not mention any causal factor”).

#### Self-perceived mate value and causal attributions

Attribution group was regressed on self-perceived mate value (SMV) in a binary logistic regression. The coefficient for SMV was once again positive: *b* = 0.50[0.08,0.91],* p* = 0.018. Thus, an increase in self-perceived mate value was associated with an increase in the probability of attributing success solely to factors of at least limited stability and generality, which lends further support to H7. The odds of making such an attribution were estimated to increase by a factor of 1.64 as self-perceived mate value increases by one point.

#### The effect of attributions on expectancy

Those who attributed success solely to factors of at least limited stability and generality were estimated to be just over half a point higher on the 0–10 expectancy scale than those who failed to attribute success (solely) to such factors: *b* = 0.54[0.003, 1.07],* t* = 2.00, *p* = 0.049—an appreciable effect. In this regression, difficulty-in-retrieval was once again estimated to have a negative effect on expectancy: *b* = −0.22[−0.42, −0.02],* t* = 2.20, *p* = 0.031. There was therefore further support for H5.

### Brief discussion

In Experiment 2, participants in each condition were asked to recall *three* examples of success or failure. As expected, fewer participants were able to recall and describe the required number of examples. As in Experiment 1, there was little to suggest that asking participants to recall success had a meaningful overall effect on expectancy. Once again, however, the hypotheses were generally supported. Participants with low self-perceived mate value apparently found it more difficult to recall success and were less likely to attribute success (solely) to factors of at least limited stability and generality. Moreover, as in Experiment 1, when participants were attempting to recall success, expectancy appeared to be negatively affected by difficulty-in-retrieval and positively affected by attributions of success to factors of at least limited stability and generality. Amongst participants attempting to recall success, ease-of-retrieval apparently had a greater effect on expectancy in Experiment 2 than in Experiment 1.

In Experiments 1 and 2, the associations between ease-of-retrieval/attributions and expectancy cannot be due to age or self-perceived mate value since those variables were statistically controlled. It might be argued, however, that they are due to another variable: the *recency* of the recalled successes. Compared to recent successes, successes that occurred in the distant past may be more difficult to recall, less readily attributed to stable and general factors and less effective in raising expectancy. If so, then any effect (on expectancy) of ease-of-retrieval or attributions is confounded with the effect of recency. This issue was addressed in Experiment 3. The hypotheses were the same except that recency was now included. Thus H5, H6 and H8 became:

- H5*:When individuals of the same age and self-perceived mate value are recalling *equally recent* (attraction) success, the greater the difficulty-in-retrieval, the lower their expectancy
- H6*:When individuals of the same age and self-perceived mate value are recalling *equally recent* (attraction) failure, the greater the difficulty-in-retrieval, the higher their expectancy.
- H8*:When individuals of the same age and self-perceived mate value experience the same ease/difficulty in recalling *equally recent* (attraction) success, those who attribute successes solely to factors of at least limited stability and generality have higher expectancy of future success than individuals who fail to attribute successes (solely) to such factors.

## Experiment 3

### Methods

#### Participants

The recruitment process and inclusion criteria were the same as in Experiments 1 and 2. Initially, 270 individuals were recruited and randomly assigned to the “recall success” *(n* = 135) or “recall failure” *(n* = 135) condition. In the “recall success” condition 129 participants (96%) and in the “recall failure” condition 127 participants (94%) completed the study. Those who did not complete the study did not submit any data and were therefore excluded from analyses. Ages ranged from 19 to 72 (M = 31.9; SD = 10.0). Reported nationalities included British (66%), North American (15%), South African (4%), Australian or New Zealander (3%) and various other nationalities, e.g. Spanish, Chinese, Pakistani and Nigerian (12%). In response to the question about gender, 51% participants selected “female” (130 participants), 48% selected “male” (122 participants), and 1% selected “other” (4 participants).

#### Procedure

The procedure of Experiment 3 was identical to the procedure of Experiment 2 except in the following respect. Participants in each condition were asked not only to recall and describe three successes/failures but also to indicate when each success/failure occurred.

### Measures

Estimated reliability was again very high for self-perceived mate value (*α* = 0.89), expectancy (*α* = 0.92) and ease-of-retrieval (*α* = 0.90), which were measured by means of the instruments used in Experiments 1 and 2.

#### Recency

Participants were asked to indicate when each success or failure occurred. A drop-down list provided 71 options (e.g. “1 year ago,” “2 years ago”… “70 years ago”). The first option was “0 (within the last year).” The “recency” score was the mean of the options selected.

### Results

Means and standard deviations of measured variables in each condition are presented in Table 7.

**Table 7:** Means and standard deviations of measured variables in Experiment 3

	“Recall Success”		“Recall Failure”	
	M	SD	M	SD
Self-Perceived Mate Value	4.19	1.14	4.21	1.16
(Attraction) Expectancy	4.61	1.65	4.53	1.93
Ease-of-retrieval	3.99	1.39	3.80	1.24
Recency	6.15	5.73	4.93	4.34

In the “recall success” condition 106 participants (82%) and in the “recall failure” condition 116 participants (91%) were able to recall and describe three examples.

#### Self-perceived mate value and ease-of-retrieval

When ease-of-retrieval was regressed on condition, self-perceived mate value, the product of condition and self-perceived mate value, age and recency, the coefficient for the interaction term was statistically significant: *b* = −0.59 [−0.88, −0.29],* t* = 3.91, *p* = 0.0001. When self-perceived mate value (SMV) was lower than 3.74, participants experienced (statistically) significantly greater difficulty-of-retrieval in the “recall success” than in the “recall failure” condition. Almost one-third of participants (30%) had SMV scores lower than 3.74. At one standard deviation below the mean of the SMV distribution (SMV = 3.08), ease-of-retrieval was estimated to be 0.75 [0.27, 1.23] of a point lower in the “recall success” than in the “recall failure” condition (*t* = 3.10, *p* = 0.002)—a moderately large effect. There was therefore further support for H1.

Self-perceived mate value was negatively associated with difficulty-of-retrieval in the “recall success” condition: *b* = −0.32 [−0.54, −0.10],* t* = 2.86, *p* = 0.005. There was therefore further support for H2. Self-perceived mate value was positively associated with difficulty-of-retrieval in the “recall failure” condition: *b* = 0.27 [0.07, 0.47],* t* = 2.68, *p* = 0.008. There was therefore support for H3.

#### The overall effect of condition on expectancy

The overall effect on expectancy of recalling success was estimated to be positive but was again very small and far from statistical significance: *b* = 0.12 [−0.24, 0.47],* t* = 0.65, *p* = 0.516.

#### Self-perceived mate value, age, ease-of-retrieval and expectancy

Expectancy was regressed on ease-of-retrieval, condition, self-perceived mate value, recency, age, the product of condition and ease-of-retrieval and the product of condition and recency. The estimated effect of self-perceived mate value on expectancy was once again positive and large: *b* = 0.94 [0.78, 1.09],* t* = 12.03, *p* < 0.0001. There was therefore further support for H4.

The coefficient for the interaction between condition and ease-of-retrieval was statistically significant: *b* = −0.70 [−0.96, −0.43],* t* = 5.17, *p* < 0.0001. In the “recall success” condition, greater difficulty-of-retrieval was associated with lower expectancy: *b* =—0.21 [−0.39, −0.03],* t* = 2.31, *p* = 0.022. There was therefore support for H5* (when individuals of the same age and self-perceived mate value are recalling equally recent (attraction) success, the greater the difficulty-in-retrieval, the lower their expectancy). The more recent the successes the higher the expectancy but the estimated effect of recency in the “recall success” condition was exceptionally small and not statistically significant: *b* =—0.01 [−0.06, 0.04],* t* = 0.39, *p* = 0.700.

In the “recall failure” condition, greater difficulty-of-retrieval was associated with *higher* expectancy: *b* = 0.49 [0.30, 0.68],* t* = 5.06, *p* < 0.0001. There was therefore support for H6* (when individuals of the same age and self-perceived mate value are recalling equally recent (attraction) failure, the greater the difficulty-in-retrieval, the higher their expectancy). Interestingly, the more distant (i.e. further in the past) the failures, the lower the expectancy: *b* =—0.06 [−0.12, −0.01],* t* = 2.18, *p* = 0.030.

#### Qualitative data and causal attributions

The authors assigned 95.3% of participants’ responses (101 out of 106) to the same attribution category (Cohen’s κ = 0.91 [0.85,0.99]). Table 8 records the number of responses assigned to each category after the discrepancies were resolved.

**Table 8:** The Number of Responses Assigned to Each (Attribution) Category in Experiment 3

Category	Number of Responses
1. All causal factors mentioned in the response have at least limited stability and generality	58
2. The response mentions an unstable or highly specific causal factor;	38
3. The response does not mention any causal factor	10
4. The stability and generality of factors mentioned in the response are unclear and the response does not fall readily into any of the other categories	0

As may be observed from Table 8, 58 responses fell into attribution group 1 (“All causal factors mentioned in the response have at least limited stability and generality”) and 48 responses fell into group 2 (“The response mentions an unstable or highly specific causal factor” or “The response does not mention any causal factor”).

#### Self-perceived mate value and causal attributions

Attribution group was regressed on self-perceived mate value (SMV) in a binary logistic regression. The coefficient for SMV was again positive: *b* = 0.98[0.51,1.44],* p* < 0.001. Thus, an increase in self-perceived mate value was associated with an increase in the probability of attributing success solely to factors with at least limited stability and generality, supporting H7. More specifically, the odds of making such an attribution were estimated to increase by a factor of 2.66 as self-perceived mate value increases by one point.

#### The effect of attributions on expectancy

When expectancy in the “recall success” condition was regressed on age, self-reported mate value, ease-of-retrieval, recency and attribution group, participants who attributed success solely to factors with at least limited stability and generality (= group 1) were estimated to be approximately a quarter of a point higher on the 0–10 expectancy scale than participants who failed to attribute success (solely) to such factors (= group 2): *b* = 0.26[−0.29, 0.81],* t* = 0.93, *p* = 0.354. The estimated effect was consistent with H8*, albeit not statistically significant. In this regression, difficulty-in-retrieval was once again estimated to have a negative effect on expectancy: *b* = −0.22[−0.40, −0.05],* t* = 2.49, *p* = 0.014. This further supports H5*.

### Brief discussion

As in Experiments 1 and 2, the mean effect on expectancy of recalling success (vs. failure) was estimated to be very small and was not statistically significant. Once again, those with low self-perceived mate value found it more difficult to recall successes than failures. In addition, lower self-perceived mate value was associated with greater difficulty-in-retrieval within the “recall success” condition and greater ease-of-retrieval within the “recall failure” condition. In the “recall success” condition, difficulty-in-retrieval was once again associated with lower expectancy. Experiment 3 controlled not only for age and self-perceived mate value but also for the recency of recalled successes. Experiment 3 therefore provides even stronger evidence that expectancy is affected by ease-of-retrieval when individuals attempt to recall success.

As was the case in Experiments 1 and 2, participants with lower self-perceived mate value were apparently less likely to attribute success to factors of at least limited stability and generality. Moreover, the impact (on expectancy) of attributing success to such factors was once again estimated to be positive. The estimated effect (approximately ¼ of a point) was however smaller than in Experiments 1 and 2 and was not statistically significant. It is possible that drawing participants’ attention to the “recency” of successes diluted the impact of attributions. For example, if Bob is reminded that his successes occurred 3, 5 and 7 years ago then an attribution to a stable and general cause (e.g. “I have an attractive personality”) may have less of an effect on his expectancy. More specifically, although Bob identifies a stable and general internal characteristic (e.g. his personality) he may also realise that (external) circumstances have changed over the years. The stable and general characteristic (his personality) may then carry less weight in influencing expectancy.

## General discussion

Recalling past success is widely thought to enhance expectations of future success (e.g. Gandhe, 2023; Harker, 2011; Schwindt, 2014). Moreover, many forms of coaching, therapy and personal development involve asking individuals to recall success (e.g. Beck, 2021; Conoley & Scheel, 2018; Ives & Cox, 2014). Experimental research, however, has yielded mixed results. In some studies, asking individuals to recall success has apparently enhanced expectancy-like variables (e.g. Austin & Costabile, 2021; Brown et al., 2016; Nelson & Knight, 2010). In others, it appears to have had little or no positive effect (e.g. Abdulla, 2021; Solms et al., 2022; Vanlede et al., 2009). One of the main aims of the present study was to investigate two (meta)cognitive factors that may account for discrepancies: ease-of-retrieval and causal attributions. Previous studies of “recall success” interventions have not simultaneously examined these factors or controlled for confounding variables. Moreover, almost all previous studies have involved students only. The present study therefore makes an important contribution.

In three experiments involving more than 700 adults across the English-speaking world participants struggling to attract “the right person” were asked to recall examples of attraction success or failure. The mean effect of recalling success (vs. failure) was estimated to be extremely small in all three experiments. However, in participants attempting to recall success, expectancy appeared to be negatively affected by difficulty-in-retrieval and positively affected by attributions of success to stable and general factors. Importantly, expectancy was associated with ease-of-retrieval and attributions even when age, self-perceived mate value and recency of success were all statistically controlled.

As difficulty-in-retrieval in recalling success increased by 1 point expectancy was estimated to drop by 1/10 to 1/5 of a point on a 0–10 scale. This is not in itself a large effect but it is important to realise that ease-of-retrieval may have a marked impact on expectancy if individuals find it *very* difficult or *very* easy to recall success. Indeed, the relative difficulty-in-retrieval experienced by many participants in the present study may go a long way towards explaining why a positive mean effect was not apparent. Comparison with the study reported by Wehr (2010) is instructive. Wehr (2010, p.471) reports that participants found it “*much easier* to retrieve exception times [i.e. successes] than exemplary problem episodes [i.e. failures]” (italics added). In the present study, the opposite was the case for a substantial percentage of participants: retrieval of success was estimated to be considerably *more difficult* than retrieval of failure. The positive mean effect of recalling success reported by Wehr (2010) and the apparent lack of such an effect in the present study may therefore be due to differences in ease-of-retrieval.

Moreover, there are reasons for thinking that participants recalling success in other studies also experienced considerably greater ease-of-retrieval than participants in the present investigation. Note that in the three experiments reported here participants were asked to recall success in a domain in which they were struggling (viz. attracting a romantic partner). On the other hand, participants in most of the studies reviewed in the introduction were asked to recall success in *any* domain (e.g. Austin & Costabile, 2021; Nelson & Knight, 2010; Snyder et al., 1996). Recalling success in a domain of one’s choosing is likely to be (considerably) easier than recalling success in a domain in which one is struggling. Somewhat paradoxically, therefore, individuals struggling in a particular domain may be more likely to experience an expectancy boost if they are asked to recall success in *another* domain and left to choose that domain.

Results of the present study also suggest that when individuals are recalling past success, expectations of future success depend partly on the factors to which the recalled success is attributed. In all three experiments participants who mentioned (only) stable and general factors (e.g. “I have an attractive personality”) reported higher expectancy on average than participants who failed to mention such factors (exclusively). The difference was statistically significant in Experiments 1 and 2 (but not in Experiment 3) and was estimated to be approximately ¼ to ¾ of a point on a 0–10 scale. Importantly, evidence of this effect was obtained even when potential confounders (e.g. self-perceived mate value and ease-of-retrieval) were statistically controlled. 

Ease-of-retrieval and attributions were themselves predicted by self-perceived mate value. That is, participants with lower self-perceived mate value tended to find it more difficult to recall examples of success and were less likely to attribute success to stable and general factors. This finding has important practical implications. Those with low self-perceived mate value are likely to be those most in need of an expectancy boost. Unfortunately, however, they may also be unlikely to benefit from a “recall success” manipulation. More specifically, their difficulty in recalling examples of success and failure to attribute success to stable/general factors may undermine the intervention.

Other studies suggest that individuals who are struggling psychologically may be unlikely to benefit from “recall success” interventions. It appears, for example, that dysphoric or depressed individuals do not (always) experience an improvement in mood after recalling positive events (Isham et al., 2022). Similarly, chronically sad individuals may not experience an increase—and may actually experience a decrease—in self-esteem after bringing to mind previous successes (Gebauer et al., 2008). Together with the results of the present study, these findings suggest that recalling success may be ineffective for those who are in most need of help.

In the present study, the question asked (“What does it say about you that you were able to recall that person/those people?”) was designed to encourage participants to attribute success to internal, stable and general factors (e.g. “I have an attractive personality”). Nevertheless, a considerable number of participants (particularly those with low self-perceived mate value) failed to make such attributions. In order for a “recall success” intervention to be effective, additional prompts may be required. In a study reported by Zunick et al. (2015), participants asked to recall success were then required to complete the following sentence stem: ‘I was able to achieve a successful performance *because I am*...’” (italics added). This prompt was apparently instrumental in raising participants’ expectancy. The present tense of “I am…” may have helped to elicit attributions of success to stable and general factors (e.g. “because I am intelligent and charismatic”). Researchers examining “recall success” interventions may wish to investigate whether other prompts are even more likely to encourage effective attributions.Limitations of the present study should be acknowledged. Although goal attainment expectancy is naturally (and commonly) assessed via self-report, the risk of demand characteristics and socially desirable responding should be considered, particularly in “recall success” conditions. There were no meaningful differences in mean expectancy scores between “recall success” and “recall failure” conditions, which suggests that “recall success” participants were not systematically inflating their scores. Nevertheless, researchers in this domain may wish to include a behavioural measure—less susceptible to response bias—such as performance on a task requiring perseverance (e.g. DiMenichi & Richmond, 2015). In the present context (romantic attraction), researchers could measure the amount of time spent on an attraction-related activity, e.g. use of a dating app. It should be noted, however, that task performance and time spent on an activity are (at best) *indirect* measures of expectancy.

The experiments in the present study were conducted in the context of interpersonal attraction. Replications or extensions should explore the relationships between the key variables (e.g. between ease-of-retrieval and expectancy) in other contexts as well. Zunick et al. (2015) found that a “recall success” intervention enhanced expectancy in the context of public speaking but did not simultaneously examine ease-of-retrieval and attributions. Researchers may therefore wish to replicate the present study in the context of public speaking. Self-perceived mate value (the facet of self-esteem most relevant in the context of attraction) would need to be replaced by a variable such as self-perceived public speaking competence (e.g. Liu, 2024).

In summary, the present study furthers our understanding of the mechanics of “recall success” interventions, at least in the context of attraction. Recalling examples of success may not be enough to raise (attraction) expectancy. How those examples are retrieved and interpreted appears to be very important. The findings of the study support Weiner’s Attribution Theory insofar as expectations of future success (apparently) depend on both the perceived stability and perceived generality of the causes of past success (Weiner, 2012). The findings of the study also support two of the tenets of Bandura’s theory of (perceived) self-efficacy: 1) “even success experiences do not necessarily create strong generalized expectations of personal efficacy” and 2) “[t]he impact of information on efficacy expectations will depend on how it is cognitively appraised” (Bandura, 1977, p.200). Results of the present study also support the theoretical distinction between “availability” and “ease-of-retrieval” (MacLeod & Campbell, 1992; Tulving & Pearlstone, 1966). Examples of success may be “available” to individuals in the sense that they are stored and “exist” in memory. However, the ease or difficulty with which they are recalled appears to influence expectancy. This is consistent with the results of other studies that have found a similar association between ease-of-retrieval and expectancy. For example, Abdulla and Woods (2022) found that the more difficulty participants experienced in generating means of goal attainment the lower their expectations of success in goal pursuit. The (meta)cognitive factors—ease-of-retrieval and attributions—therefore deserve greater attention in research on “recall success” interventions. Finally, it should be clear that much more research is required in order to clarify when, how and for whom recalling success is (and is not) effective.

## Data Availability

Data are available on request from the corresponding author.
